# Effects of the particle of ground alfalfa hay on the growth performance, methane production and archaeal populations of rabbits

**DOI:** 10.1371/journal.pone.0203393

**Published:** 2018-09-17

**Authors:** Siqiang Liu, Mei Yuan, Dingxing Jin, Zhisheng Wang, Huawei Zou, Lizhi Wang, Bai Xue, De Wu, Gang Tian, Jingyi Cai, Tianhai Yan, Quanhui Peng

**Affiliations:** 1 Institute of Animal Nutrition, Key Laboratory of Bovine Low-Carbon Farming and Safety Production, Sichuan Agricultural University, Wenjiang, Chengdu, PR China; 2 Agri-Food and Biosciences Institute, Hillsborough, Co. Down, United Kingdom; The University of Sydney, AUSTRALIA

## Abstract

The world's annual output of rabbits is over 1.2 billion, therefore this sector is also one of the sources of greenhouse gases in livestock production. One hundred-twenty New Zealand rabbits were allocated into four treatments, five replicates in each treatment and six rabbits in each replicate to examine the effect of grinding alfalfa hay to different sizes on growth performance, methane production and cecal archaeal populations. The particle sizes of the alfalfa meal in the four treatment diets were 2500, 1000, 100 and 10 μm, while the other ingredients were ground through a 2.5 mm sieve. The average daily gain (ADG) and average daily feed intake (ADFI) increased (*P*<0.001) as the particle size decreased, but the feed conversion ratio (FCR) was not affected (*P* = 0.305). The digestibility of neutral detergent fiber (NDF) (*P* = 0.006) and acid detergent fiber (ADF) (*P*<0.006) increased while the greatest digestibility of crude protein (CP) was obtained in 1000 um group (*P* = 0.015). The rabbits produced more methane (CH_4_, L/kgBM^0.75^/d) with decreasing alfalfa particle size (*P*<0.001). The molar proportion of acetic acid and propionic acid decreased (*P*<0.001) at the cost of butyric acid (*P*<0.001). The greatest villus height:crypt depth ratio were obtained in 1000 μm group, and the decrease in the alfalfa hay particle size decreased the jejunum and ilem villus height:crypt depth ratio (*P*<0.05). The gastric muscular and mucosal thickness decreased with decreasing alfalfa particle size (*P*<0.05). Archaea diversity decreased with decreasing alfalfa particle size, and the relative abundance of genus *Methanobrevibacter* increased (*P*<0.001) while the genus *Methanosphaera* decreased (*P*<0.001). It is concluded that a finer particle size favors the growth of genus *Methanobrevibacter*, which produces more methane but promotes the growth performance of rabbits.

## Introduction

The rabbit is an herbivorous animal, and due to its nutritional and physiological characteristics, it depends on fiber to maintain the health of its gastrointestinal tract. Fibrous feeds not only provide energy in the form of short-chain fatty acids produced by fermentation by microorganisms in the rabbit cecum, but they also stimulate gastrointestinal peristalsis and increase the speed of chyme efflux, which are physical factors that prevent digestive dysfunction [1–2].

Alfalfa is commonly a major ingredient in rabbit diets, and it is considered a balanced source of fiber to meet the requirements of the animal, accounting for approximately one-third of commercial feeds [3–4]. The physical structure of the fiber, especially the particle size, is an important feature that has an important effect on the rabbit digestion process: particle size affects the retention time in the gastrointestinal tract [5–6], feed intake [7–8] and nutrient digestibility [2–9]. With the continuous advancement in the development of feed resources, feed nutrient components have become increasingly diverse and complex, and crushing raw materials facilitates more uniform mixing of the components. However, most previous studies of feed stuff particles have focused on sizes larger than 1 mm [10–12]; the effect of fiber particle size less than 1 mm has mostly been ignored.

Methane (CH_4_) is the second most important greenhouse gas due to its high global warming potential (23 times greater than that of carbon dioxide) and its emission rate [13]. Herbivorous monogastric animals produce little methane, but due to the large number of such animals, their total production can be huge. Methane production is the result of a series of metabolic interactions among various microbial populations, among which methanogenic archaea make important contributions (up to 22% of total microbial RNA in the rabbit cecum), and *Methanobrevibacter* sp. have been highlighted [14–15]. Although many studies involve cecal fermentation, the study of methane production by rabbit is limited [16–17]. Several previous investigations mainly conducted were *in vitro* experiments [18–21]. Therefore, the objective of this work was to study the effects of the particle size of ground alfalfa hay on growth performance as well as cecum fermentation characteristics, enteric methane emissions, and cecal archaeal populations.

## Materials and methods

This study was approved by the Animal Policy and Welfare Committee of the Agricultural Research Organization of Sichuan Province, China, and the rabbits were handled according to the principles for the care of animals used for experimentation.

### Animals and housing

One hundred twenty New Zealand rabbits (60 male and 60 female) had been weaned (35 d of age) with an average weight of (946 ± 82 g) were used for the experiment. The rabbits were divided into four dietary treatment groups (5 replicates per group and six rabbits per replicate, and the rabbits were kept in 3 pairs with 2 rabbits per cage). Rabbits were housed in the same building in flat-deck cages measuring 600 × 250 × 330 mm and were provided access to feed and water *ad libitum*. All rabbits were kept under controlled environmental conditions (the room temperature was between 15 and 25°C).

### Experimental diets

A common experimental diet was formulated for all the treatments according to the recommendations [22–23]. At first, alfalfa meal with particle sizes of 2500, 1000, 100 and 10 μm were produced. The rest of the ingredients were milled through a 2.5-mm grinder screen. After all the ingredients were ready, mix and granulate. The diameter of the pellets was 3 mm, and the ingredients and chemical compositions of the experimental diets are shown in Table 1. Neither feed nor drinking water was medicated with antibiotics, but a coccidiostatic (robenidine) was provided in the feed. The laser particle size distribution instrument with JL-1197(Jingxin, Chengdu, China)was used to test specific surface area of alfalfa meal with particle sizes of 2500, 1000, 100 and 10 μm, and average sizes of 100 and 10 μm. The average sizes of alfalfa meal with particle sizes of 2500 and 1000 μm were analyzed using ASAE S319.3 standard [24] particle size analysis method(S1 Table).

**Table 1:** Ingredients of experimental diets and chemical composition.

Ingredient, % as fed	Particle size (μm)			
	2500	1000	100	10
Corn	23.80	23.80	23.80	23.80
Wheat bran	29.10	29.10	29.10	29.10
Soybean oil	1.00	1.00	1.00	1.00
Soybean meal	8.50	8.50	8.50	8.50
alfalfa meal	35.20	35.20	35.20	35.20
Calcium carbonate	0.05	0.05	0.05	0.05
Calcium bicarbonate	0.59	0.59	0.59	0.59
L−Lysine	0.10	0.10	0.10	0.10
Choline chloride	0.15	0.15	0.15	0.15
DL-methionine	0.13	0.13	0.13	0.13
Sodium chloride	0.40	0.40	0.40	0.40
Mineral and vitamin premix*	1.00	1.00	1.00	1.00
Chemical composition, g/kg DM				
DM,%	90.68	90.45	90.50	90.78
Gross energy, MJ/kg of DM	16.76	16.72	16.84	16.75
Crude protein,%	15.10	15.58	16.26	16.48
Ether extract,%	3.91	3.43	3.90	3.66
Neutral detergent fiber,%	28.98	29.03	29.25	28.26
Acid detergent fiber,%	15.85	17.78	14.68	15.73
Ash,%	6.76	6.91	6.71	6.83

* Premix composition (by kg diet): Vitamin A, 8000 IU; Vitamin D_3,_ 1000 IU; Vitamin E, 2.5 mg; Vitamin K, 30.5 mg; Vitamin B_6_, 0.6 mg; Vitamin B_12_, 0.003 mg; Vitamin B_1_, 0.2mg; Vitamin B_2_, 1.6 mg; Folic acid, 0.05 mg; Nicotinic acid, 3.5 mg; Cu, 10mg; Zn, 50mg; Mn, 20mg; Fe, 50mg; Se, 0.1 mg; I, 0.5 mg; Robenidine, 100 mg.

### Growth trial

The growth tiral lasted 42 days, and the growth performance average daily feed intake (ADG), average daily feed intake (ADFI) and feed conversion ratio (FCR) of the rabbits were calculated on the first 21 days, the later 21 days and at the whole 42 days experimental period. The FCR was calculated as the ratio of ADFI to ADG.

### Digestion and metabolism trial

Fecal apparent digestibility of day matter (DM), crude protein (CP), neutral detergent fiber (NDF) and acid detergent fiber (ADF) were determined in 24 New Zealand rabbits (6 per diet treatment) weighing 3.05 ± 0.02 kg. After a 3-d period of adaptation to metabolism cages, the daily feed intake (*ad libitum* access) and total fecal output were recorded for each rabbit over a 4-d period (cecotrophy was allowed during the digestion trial). Under the metabolic cage, there was a wire filter for feces collection, and a plastic film below the filter for urine collection. The total daily fecal output of each rabbit was thoroughly mixed, quantitatively transferred into a pre-weighed plastic container and weighed, and the feces were stored at -20°C and later dried at 65°C for 48 h and ground with a 1-mm screen. Urine was collected daily in sealed plastic containers placed below the metabolic crates and then transferred into a graduated plastic container containing 20% sulfuric acid (H_2_SO_4_). A 10% aliquot of the total daily urine output was removed each day and stored in a freezer (-20°C) until analysis.

### Methane emission measurements

The rabbits were weighted and transferred into indirect, open-circuit respiration chambers with six rabbits per chamber. Before the study, the rabbits were acclimated to the chambers to minimize stress, and they were then housed in the chambers for 4 days to measure oxygen consumption and the outputs of carbon dioxide and CH_4_. CH_4_ values reported were the 4-d average for individual rabbit. The total volume of 6 m^3^ (2.5 m long, 1.5 m wide, and 1.6 m high) was ventilated by suction pumps set at range of 16 to 20 m^3^/h, allowing a slight negative pressure within the chambers. Temperature and humidity control were achieved with air conditioning units set at 16 ± 1°C and 60 ± 10% relative humidity, respectively. The exhaust air was removed from each chamber separately for measurement of volume, temperature, humidity, and pressure. The CH_4_ concentrations in the air into and out of each individual chamber were measured every 10 min (the interval for each chamber and the ambient air at 2 min) using a MGA3000 Multi-Gas Analyzer (ADC Gas Analysis Ltd., Hoddesdon, Hertfordshire, United Kingdom). The analyzer was calibrated weekly using oxygen-free N_2_ (zero gas) and a known quantity of CH_4_ (span gas). The flow measurement systems were checked before and immediately after the experiment by releasing analytical grade CH4 into the chambers, by determining the recovery of CH_4_. The purpose of the calibrations was to ensure a recovery rate of CH_4_ at a range of 97 to 103%. The concentration of O_2_, CO_2_ and CH_4_ were analyzed using gas chromatography. The rabbits were housed in metabolic crates, which were individually placed in each chamber with 3 cages per chamber that each cage 2 rabbits. Each cage contained a feed bin, drinking water container, and separate trays to collect feces and urine. The chambers were opened once daily at 0800 h to deliver enough experimental diets and water and collect feces and urine. Methane energy (CH_4_E, kJ/d) was calculated according to Blaxter et al. [25] as CH_4_E = 39.54 kJ/L*CH_4_ (L/d).

### Histological procedures and cecal trial

At the end of the growth trial, 24 animals were slaughtered (6 per diet), by cervical dislocation 1 h before dark (1900 h) to avoid soft feces excretion. Once slaughtered, the cecum of each rabbit was excised, and the pH of the cecal content was directly measured inside the organ with a glass electrode pH meter. Cecal contents were immediately sampled, frozen in liquid nitrogen and stored at -80°C for subsequent analyses and the remaining portion was stored at -20°C for volatile fatty acid (VFA) ammonia nitrogen (N-NH_3_), and microbial crude protein (MCP) assay.

The gut fragments (jejunum and ileum) and stomach from six rabbits per group were fixed by immersion in 4% (w/v) neutral formalin for 24 h at 48°C, and after fixation, the fragments were dehydrated and embedded in paraffin. Tissue sections (4 mm thick) were de-waxed and stained using the routine hematoxylin-eosin technique for morphological investigations. Five slides containing jejunum, ileum and stomach cross sections were prepared for each sample and viewed at 40× magnification under a light microscope (BX40; Olympus, Hamburg, Germany) to measure the villus height and crypt depth from 24 intact jejunum and ileum, and mucosal and muscular thickness of stomach.

### Analytical methods

All chemical analyses were conducted in triplicate. The procedures of the AOAC (2000) [26] were used to determine DM (method 934.01), CP (method 984.13). The method described by Van Soest et al. [27] was used to determine NDF and ADF, both NDF and ADF were corrected by its ash content. The energy concentration of the feed, feces, urine and leftovers were measured in a bomb calorimeter (Parr 6300 Calorimeter, Moline, IL, USA). A total of 24 feces samples, 24 urine samples, 8 feeds samples and 8 leftovers (leftovers were mixed and sampled each 21 days) were determined. The digestible energy (DE) and metabolizable energy (ME) was calculated using the following equations:

DE=grossenergy(GE)‑fecalenergy(FE);

$$\mathrm{ME}=\mathrm{GE}‑\mathrm{FE}‑\mathrm{urinary}\;\mathrm{energy}\;\left(\mathrm{UE}\right)‑\mathrm{methane}\;\mathrm{energy}\;\left({\mathrm{CH}}_{4}E\right).$$

The thawed samples of the cecal contents were centrifuged (2,500 × g) at 0°C for 10 min and the N-NH_3_ concentration of the supernatant was measured using a spectrophotometer according to the method of Weatherburn et al. [28].

Samples for VFA determination were distilled with sodium tetraborate solution (2.5%) collected in boric acid solution (1%) and valorated with hydrochloric acid (0.05 M) and a color indicator. Cecal contents (1 g) were mixed with 2 mL of distilled water in a screw-capped tube, and the suspension liquid was centrifuged (12,000 × g) at 4°C for 10 min. The supernatant (2 mL) were mixed with 0.2 mL of metaphosphoric acid and centrifuged for 30 min at 4°C. Aliquots of the supernatants (1 μL) were analyzed by a Varian CP-3800 gas chromatograph (Agilent Technologies, Santa Clara, CA, USA) and a flame ionization detector was used at an oven temperature between 100°C and 150°C. The polyethylene glycol column was operated with highly purified N_2_ as the carrier gas at 1.8 mL/min. The lower detectable limit for all volatile fatty acids (VFAs) was 0.1 mM. Trichloroacetic acid (TCA) precipitation was used to determine cecal MCP: Cecal contents (1 g) were mixed with 3 mL of physiological saline in a screw-capped tube, and after mixed, centrifuge 10 min at 2000 × g, 4°C. Deprecipitate, supernatant 15000 × g 20min, remove the supernatant, add 1mL 5% TCA to the precipitate, then 10000 × g 10min, dissolve the precipitate with NaOH (1M), dilute to 25mL with distilled water were assayed spectrophotometrically (UV-120-02, Shimadzu, Tokyo, Japan) at OD_260nm_ and OD_280nm_. MCP (mg/mL) = (1.45 × OD_260nm_-0.47 × OD_280nm_) × dilution factor.

### Bioinformatics analyses (DNA extraction, sequencing)

Total genomic DNA was extracted from the samples using a DNeasy PowerSoil Kit (Qiagen, Valencia, CA, USA) according to the manufacturer’s instructions. The purified DNA was amplified in triplicate by PCR using universal bacteria archaea 16S rRNA gene (variable region V4) forward Arch 516F (5'-TGYCAGCCGCCGCGGTAAHACCVGC-3') and reverse Univ 806R (5'-GGACTACHVGGGTWTCTAAT-3') primer pairs [29]. The PCR mixture (25μl) contained 1 × PCR buffer, 1.5 mM MgCl_2_, each deoxynucleoside triphosphate at 0.4 μM, each primer at 1.0 μm, 0.5 U of KOD-Plus-Neo (Toyobo, Tokyo, Japan) and 10 ng of template DNA. The PCR amplification program consisted of initial denaturation at 94°C for 1 min followed by 30 cycles (denaturation at 94°C for 20 s, annealing at 54°C for 30 s, and elongation at 72°C for 30 s), and a final extension at 72°C for 5 min. Three replicates of the PCR reactions for each sample were combined and the PCR products were purified using Gel Extraction Kit (Omega Bio-Tek, USA). DNA was sequenced using a MiSeq Reagent Kit v2 and the MiSeq System (Illumina Inc., San Diego, CA, USA) and the sequences were analyzed using QIIME software (version 1.8.0) [30]. To maintain the Phred quality score of the reads, low-quality sequences were trimmed using Trimmomatic and Usearch before assembly with the paired-end assembler [31]. UPARSE was used to cluster the sequences into OTUs (operational taxonomic units) as well as choose the representative sequence of each OTU at 97% similarity [32] followed by the removal of chimeras and singletons by UCHIME [33]. Taxonomies were assigned using the Silva database and the uclust classifier in QIIME [34] and the non-archaeal OTUs were removed. Four alpha diversity indices (Simpson, Shannon-Wiener, Chao1 and phylogenetic distance) were calculated. Principal component analysis (PCA) was applied to reduce the dimensions of original community data. The OTU table, rarefaction dilution curves, network analysis, heat map analysis and beta diversity analysis were performed using R programming tools (version 3.3.0).

### Statistical analysis

The MIXED model (SAS 9.3 Institute Inc., Cary, NC, USA) was used to determine the effects of alfalfa meal particle size on growth performance, nutrient digestibility, intestinal development morphology, cecal fermentation characteristics and archaeal populations. The model used for the analysis was y = μ + t_i_ +r_k_+ e_ijk_, where y is the dependent variable; μ is the population mean for the variable; t_i_ is the fixed influence of alfalfa meal particle size, r_k_ is the random effect of animal within treatments, and e_ijk_ is the random error related to the observation ijk. Tukey-Kramer multiple comparison tests were performed after differences were detected. Differences between means with *P*<0.05 were accepted as statistically significant differences.

## Results

The effect of alfalfa meal particle size on the growth performance of rabbits is shown in Table 2. The final body weight of the 2500 μm group was lower than that of the other three groups (*P* = 0.013). During the first 21 days of this experiment, the ADFI of the 100 and 10 μm groups were greater than group 2500 and 1000 μm (*P<*0.001). The ADG of the 100 and 10 μm groups were greater than those of the 2500 and 1000 μm groups (*P*<0.001). The FCR of the 2500 μm group was greater than that of groups 100 and 10 μm (*P =* 0.013).

**Table 2:** Effect of alfalfa meal particle size on the growth performance of rabbits.

**Parameter**	**Particle size (μm)**				**SEM**	*P* **value**
	2500	1000	100	10		
Initial weight(g)	945.42	959.00	938.60	947.17	8.817	0.904
Final weight(g)	2145.63^b^	2343.87 ^a^^b^	2358.60 ^a^	2369.48 ^a^	29.589	0.013
1-21d						
ADFI(g/d)	79.63^b^	80.64^b^	91.43^a^	88.70^a^	1.301	<0.001
ADG (g/d)	24.44^b^	26.83^b^	30.03^a^	29.70^a^	0.369	<0.001
FCR	3.31^a^	3.21^a^^b^	3.06^b^	3.05^b^	0.035	0.013
21-42d						
ADFI(g/d)	117.35^b^	123.98^a^^b^	129.82^a^	130.66^a^	1.375	<0.001
ADG (g/d)	31.70^b^	34.52^a^^b^	33.79^a^^b^	35.18^a^	0.440	0.023
FCR	3.82	3.67	3.95	3.73	0.052	0.259
0-42d						
ADFI(g/d)	98.49^b^	102.31^b^	110.62^a^	109.68^a^	1.131	<0.001
ADG(g/d)	28.07^b^	30.68^a^	31.91^a^	32.44^a^	0.304	<0.001
FCR	3.57	3.44	3.50	3.39	0.048	0.305

ADFI, average daily feed intake; ADG, average daily gain; FCR, feed conversion ratio.

^a,b^Different superscripts in the same row indicate significant differences (*P*≤0.05).

In the later 21 days, the ADFI of the rabbits increased with decreased alfalfa meal particle size. The rabbits in the 100 and 10 μm groups showed greater ADFI (*P*<0.001). The ADG of the 10 μm group was greater than that of group 2500 μm (*P* = 0.001), but there was no difference among the 2500, 1000 and 100 μm groups. The FCR did not vary with the variation in alfalfa meal particle size (*P* = 0.259).

During the entire experimental period, the ADFI of the 100 and 10 μm groups was greater than those of the 2500 and 1000 μm groups (*P<*0.001). The ADG of groups 1000, 100 and 10 μm were greater than that of group 2500 μm (*P<*0.001). Furthermore, the FCR of the rabbits in all four treatments were similar throughout the entire study period (*P* = 0.305).

Table 3 shows the effect of alfalfa meal particle size on the apparent digestibility of nutrients in rabbits. The CP digestibility of the 2500 μm group was lower than that of group 1000 μm (*P* = 0.015), and no difference was obtained among the 1000, 100 and 10 μm groups. The NDF digestibility of the 2500 and 1000 μm groups were lower than that of group 10 μm (*P* = 0.003), and the ADF digestibility group 2500 μm of were lower than that of groups 1000, 100 and 10 μm (*P* = 0.006).

**Table 3:** Effect of alfalfa meal particle size on digestibility of nutrients in rabbits.

**Parameter**	**Particle size (μm)**				**SEM**	*P* **value**
	2500	1000	100	10		
Body weight (kg) (kg)	3.01	3.11	3.03	3.04	0.024	0.450
DM (%)	66.45	65.06	63.63	63.39	0.760	0.480
CP (%)	68.96^b^	74.74^a^	70.83^a^^b^	70.80^a^^b^	0.692	0.015
NDF (%)	24.46^b^	25.14^b^	28.61^a^^b^	30.51^a^	0.730	0.003
ADF (%)	16.25^b^	19.65^a^	20.03^a^	20.18^a^	0.499	0.006

DM, dry matter; CP, crude protein; NDF, neutral detergent fiber; ADF, acid detergent fiber.

^a,b^Different superscripts in the same row indicate significant differences (*P*≤0.05).

The effect of alfalfa meal particle size of on the nitrogen and energy metabolism of rabbits is presented in Table 4. The amount of ingested nitrogen of the 2500 and 10 μm groups was lower than that of the 10 μm group (*P* = 0.001). The fecal nitrogen of group 1000 μm was lower than that of group 10 μm (*P* = 0.032). The urinary nitrogen of group 2500 μm was greater compared to group 100 and 10 μm (*P* = 0.001). There were no treatment effects on nitrogen deposition (*P* = 0.249). The total energy intake of groups 2500 and 1000 μm were greater than that of 10 μm (*P* = 0.006) (Table 4). Fecal energy increased by 25.41% in group 2500 μm compared with group 10 μm (692.59 vs. 868.58 kJ/d, *P* = 0.003), and the urine energy of groups 2500 and 1000 μm was lower than that of group 10 μm (*P*<0.001). In addition, the urine energy of group 2500 μm was lower than that of group 100 μm (*P<*0.001). The methane energy values of groups 2500 and 1000 μm were lower than those of groups 100 and 10 μm, and the value for group 2500 μm was lower than that of group 1000 μm (*P*<0.001).

**Table 4:** Effect of alfalfa meal particle size on the nitrogen and energy metabolism of rabbits.

**Parameter**	**Particle size (μm)**				**SEM**	*P* **value**
	2500	1000	100	10		
Nitrogen metabolism						
FN(g/d)	0.91^a^^b^	0.81^b^	1.00^a^^b^	1.24^a^	0.060	0.032
UN(g/d)	0.50^b^	0.61^a^^b^	0.73^a^	0.67^a^	0.024	0.001
DN (g/d)	2.06	2.46	2.39	2.46	0.058	0.039
RN(g/d)	1.57	1.86	1.66	1.78	0.054	0.249
Nitrogen metabolism						
FE(kJ/d)	692.59^b^	755.59^a^^b^	797.42^a^^b^	868.58^a^	18.924	0.003
UE (kJ/d)	47.65^c^	53.84^b^^c^	57.30^a^^b^	62.33^a^	1.353	<0.001
DE(kJ/d)	1393.60	1393.18	1396.91	1475.88	23.498	0.550
CH_4_E(kJ/d)	2.22^c^	3.97^b^	4.94^a^	5.01^a^	0.240	<0.001
ME (kJ/d)	1374.29	1402.68	1369.61	1443.55	12.120	0.109

FN, fecal nitrogen; UN, urinary nitrogen; DN, digestible nitrogen; RN, retention nitrogen; FE, fecal energy; UE, urine energy; DE, digestible energy; CH_4_E, methane energy, which was calculated according to Blaxter and Clapperton (1965); ME, metabolizable energy.

^a,b,c^Different superscripts in the same row indicate significant differences (*P*≤0.05).

Effect of alfalfa meal particle size on methane emission by rabbits is displayed in Table 5, and the results showed that the methane emissions (L/d) of groups 2500 and 1000 μm were lower than those of groups 100 and 10 μm. Moreover, the value of the 2500 μm group was lower than that of the 1000 μm group (*P*<0.001). The methane production per unit metabolic weight of groups 2500, 1000 and 100 μm was lower than that of 10 μm group, and the production by the 2500 μm groups was lower than that of the 1000 and 100 μm group (*P*<0.001). The methane outputs per unit of DM intake of groups 2500 and 1000 μm were lower than those of groups 100 and 10 μm, and that of group 2500 μm was lower than group 1000 μm (*P*<0.001). CH_4_E/GE, CH_4_E/DE and CH_4_E/ME exhibited the same trend per unit of DM intake that groups 2500 μm and 1000 μm were lower than groups 100 and 10 μm, and group 2500 μm was lower than 1000 μm (*P*<0.001).

**Table 5:** Effect of alfalfa meal particle size on methane emission in rabbits.

**Parameter**	**Particle size (μm)**				**SEM**	*P* **value**
	2500	1000	100	10		
BW (kg)	2.50	2.56	2.52	2.51	0.015	0.570
DMI (g/d)	136.93	140.46	138.54	142.72	1.101	0.288
Methane production						
L/d	0.06^c^	0.10^b^	0.12^a^	0.13^a^	0.006	<0.001
L/kg BM^0.75^/d	0.03^c^	0.05^b^	0.06^b^	0.06^a^	0.003	<0.001
L/kg DMI	0.41^c^	0.71^b^	0.90^a^	0.89^a^	0.042	<0.001
CH_4_E/GE^b^ (%)	0.11^c^	0.19^b^	0.23^a^	0.21^a^	0.010	<0.001
CH_4_E/DE^c^(%)	0.16^c^	0.28^b^	0.36^a^	0.34^a^	0.017	<0.001
CH_4_E/ME^d^ (%)	0.17^c^	0.30^b^	0.37^a^	0.36^a^	0.017	<0.001

BW, Body weight; DMI, Dry matter intake; CH_4_E, methane energy, GE, gross energy; DE, digestible energy; ME, metabolizable energy.

^a,b,c^Different superscripts in the same row indicate significant differences (*P*≤0.05).

Table 6 shows the effect of alfalfa meal particle size on cecal fermentation characteristics. NH_3_-N concentration in group 2500 μm was greater than that in group 10 μm (*P* = 0.003). The molar proportions of acetic acid of groups 2500 and 1000 μm were lower than those of groups 100 and 10 μm (*P*<0.001). The molar proportion of propionic acid of the 2500 μm group was lower compared with that of the other three groups (*P*<0.001). The molar proportions of butyric acid of group 2500 μm was greater than that of groups 100 and 10 μm, and the value of group 1000 μm was greater than that of group 10 μm (*P*<0.001). There were no differences in pH, TVFA and MCP was obtained (*P*>0.05).

**Table 6:** Effect of alfalfa meal particle size on cecal fermentation characteristics of rabbits.

**Parameter**	**Particle size (μm)**				**SEM**	*P* **value**
	2500	1000	100	10		
pH	5.99	5.91	5.82	5.81	0.057	0.675
NH_3_-N(mmol/L)	23.43^a^	20.65^a^^b^	20.43^a^^b^	16.79^b^	0.702	0.003
TVFA (mmol/L)	49.05	48.71	56.18	54.64	1.808	0.460
Acetic acid (% total VFA)	77.64^b^	77.85^b^	79.16^a^	79.51^a^	0.206	<0.001
Propionic acid (% total VFA)	4.74^b^	5.25^a^	5.04^a^	5.10^a^	0.046	<0.001
Butyric acid (% total VFA)	17.62^a^	16.90^a^^b^	15.81^b^^c^	15.40^c^	0.216	<0.001
MCP(mg/mL)	6.17	6.56	6.26	6.39	0.141	0.414

NH_3_-N, ammonia nitrogen; TVFA, total volatile fatty acid; MCP, microbial protein.

^a,b,c^Different superscripts in the same row indicate significant differences (*P*≤0.05).

Table 7 presents the histological evaluation of the small intestine of rabbits. Compared with group 1000 μm, the villus height of the jejunum of groups 2500, 100 and 10 μm decreased by 25.85%, 17.89% and 14.25% (*P*<0.001), respectively, and the highest and lowest crypt depths were observed in groups 100 (108.85 μm) and 10 μm (92.55 μm) (*P =* 0.004). The villus height:crypt depth ratio of the jejunum in group 1000 μm was greater than the three other groups (*P* = 0.010) (S1 Fig).

**Table 7:** Effect of alfalfa meal particle size on gasto-intestinal morphology of rabbits.

**Parameter**	**Particle size (μm)**				**SEM**	*P* **value**
	2500	1000	100	10		
Jejunum						
Villous height (μm)	507.53^b^	638.73^a^	524.49^b^	459.07^b^	13.696	<0.001
Crypt depth (μm)	104.00^a^^b^	103.17^a^^b^	108.85^a^	92.55^b^	1.650	0.004
Villus height: crypt depth	5.05^b^	6.30^a^	5.18^b^	5.22^a^^b^	0.151	0.010
Ileum						
Villous height (μm)	648.03^b^	844.09^a^	733.27^b^	690.20^b^	13.428	<0.001
Crypt depth (μm)	123.87^a^	121.46^a^^b^	110.19^b^^c^	108.24^b^	1.839	0.003
Villus height: crypt depth	5.42^b^	7.39^a^	6.95^a^	6.75^a^	0.168	<0.001
Mucosal thickness (μm)	925.51^a^	867.14^b^	825.31^b^	745.90^c^	9.691	<0.001
Muscular thickness (μm)	721.72^a^	668.31^a^^b^	653.16^a^^b^	616.29^b^	13.026	0.036

^a,b,c^Different superscripts in the same row indicate significant differences (*P*≤0.05).

The particle size of alfalfa meal affected the villus height of the ileum, and the maximum villus heights were also observed in group 1000 μm and were greater than those of the other three groups (*P*<0.001). The crypt depth of the ileum was greater in group 2500 μm than in groups 100 and 10 μm (*P* = 0.003). Rabbits in group 2500 μm had the lowest villus: crypt ratio compared with the other three groups (*P*<0.001). The mucosal and muscular thickness of stomach decreased with the decrease of alfalfa particle size (*P*<0.05), and more degeneration and necrosis of mucosal epithelial cells, gastric inflammatory cell infiltration, and muscular myometrial edema were observed more frequently in 100 and 10 μm groups compared with 2500 and 1000 μm groups (S2 Fig).

**Fig 1:**
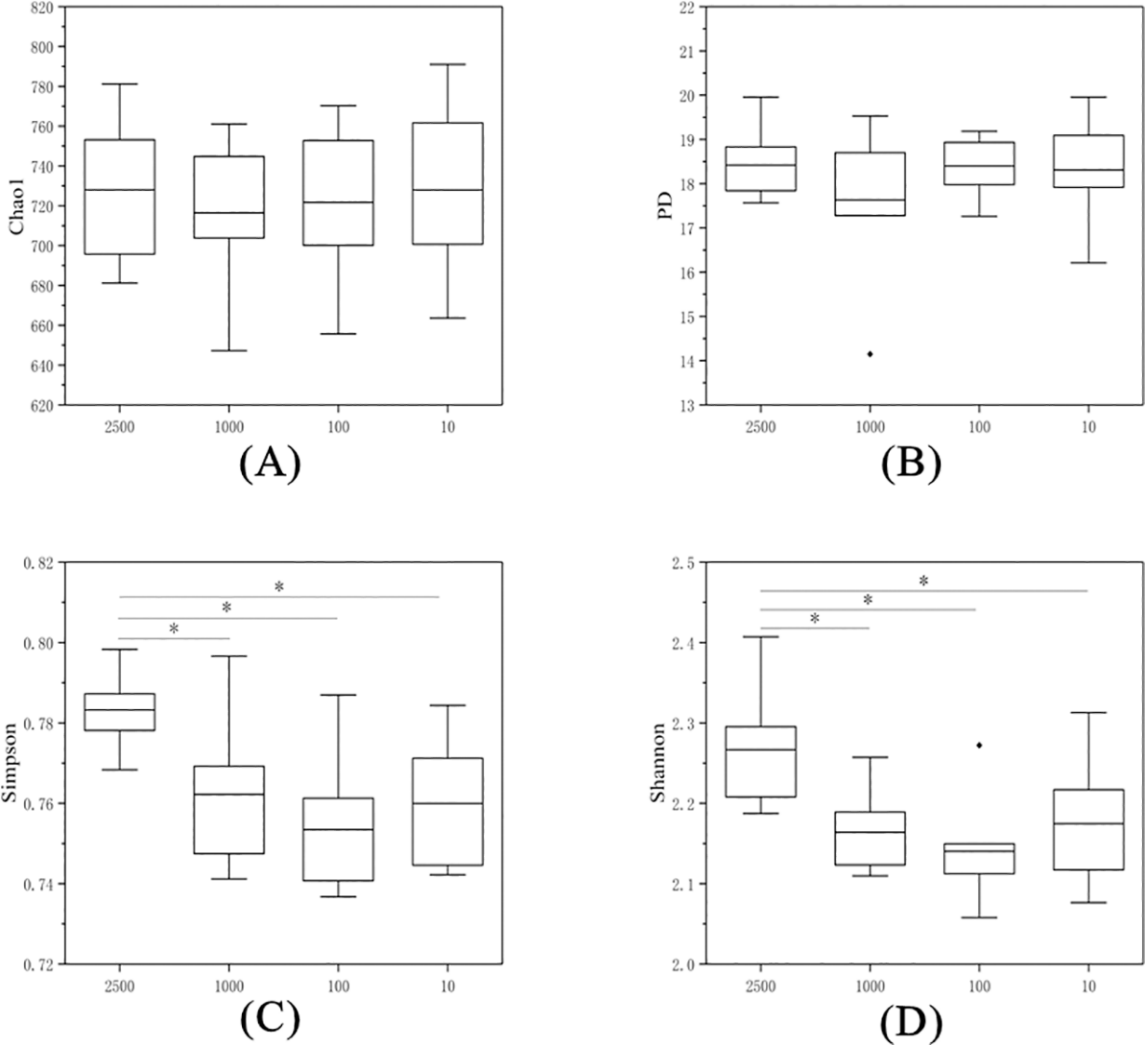
Archaea alpha diversity indices. (A) Chao1, (B) phylogenetic distance, (C) Simpson, and (D) Shannon index values of the cecal contents of rabbits. The samples were labelled 2500, 1000, 100, 10 according to different particle size of ground alfalfa hay. There are six replicates in each group.

Rarefaction curves produced from archaeal sequences are shown in S3 Fig. To further determine the important archaea, we identified the most abundant OTUS in the tested samples. In total, 12930 archaeal sequences were produced, and the sequences were clustered into 2246 OTUs based on 97% sequence similarity. The alpha diversity index values (Chao1, phylogenetic distance, Simpson and Shannon) of microbes from the cecal contents of the rabbits in each group are presented in Fig 1. The Chao1 index and the phylogenetic distances index did not differ among the four treatments, but the Simpson and Shannon indices of group 2500 μm were greater (*P*<0.05) than those of the other three groups.

**Fig 2:**
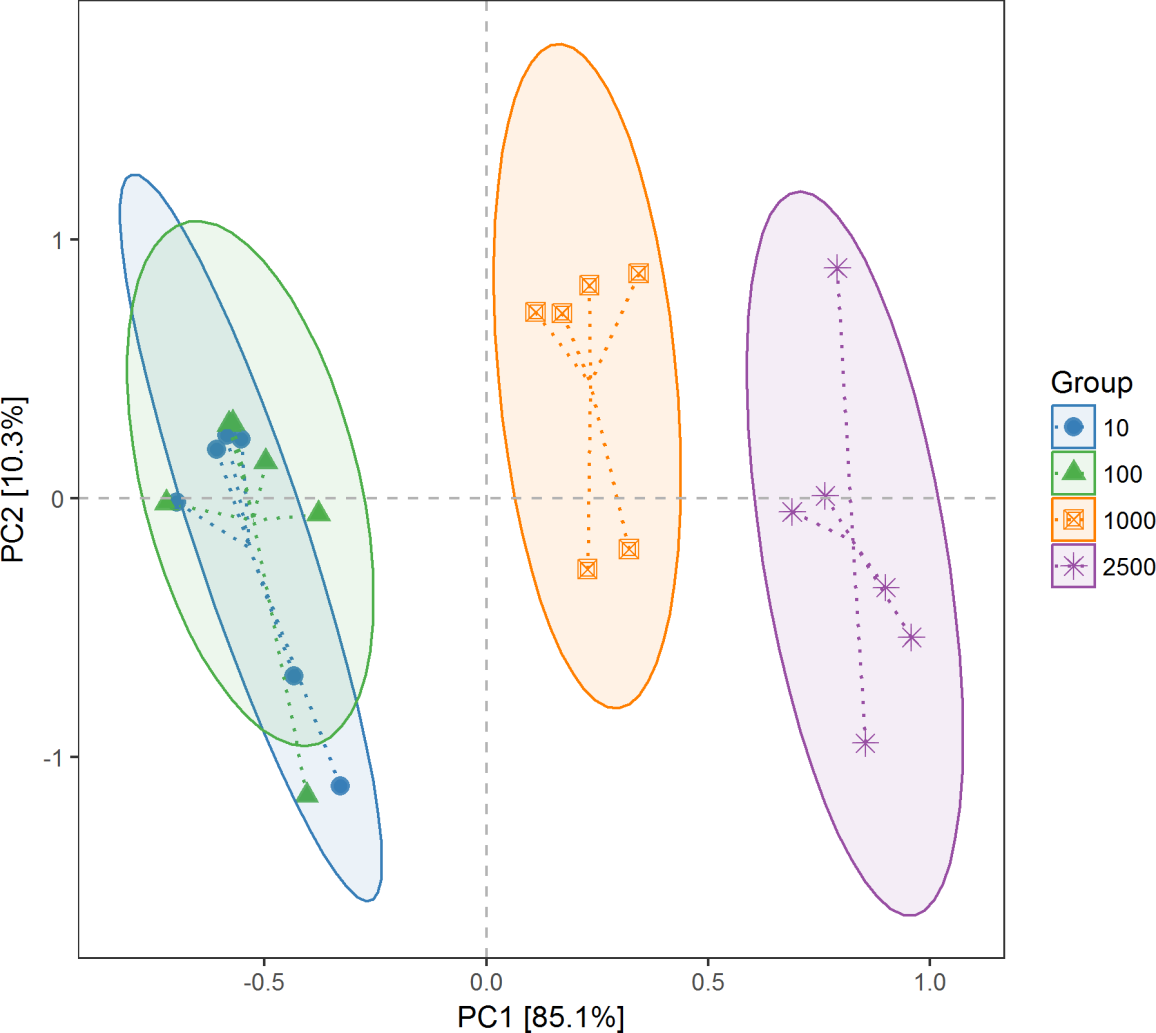
Principal component analysis (PCA) of archaeal operational taxonomic units scores plot generated by a weighted UniFrac analysis at the 97% similarity level from 24 cecal contents of rabbits. There are six replicates in each group.

The PCA plot, which was based on the relative abundance of the OTUs, revealed a separation of the different treatments into two clusters, which accounted for 85.1% and 10.3% of the total variation (Fig 2). The smaller the distance between points, the more similar the community structure of the two samples. No difference was observed between groups 2500 and 1000 μm for the archaeal community composition. When the alfalfa meal particle size decreased to 100 μm, the microbial composition completely changed compared with 2500 and 1000 μm groups, but when the particle size decreased from 100 to 10 μm, the microbial composition remained unchanged. These results were also reflected in a heatmap of genus abundance (S4 Fig).

A genus-level analysis performed on cecum archaeal populations of rabbits is shown in Table 8. The taxonomy-based analysis of the 24 samples showed that there were five genera in the archaeal communities of cecum contents. The relative abundance of genera *Methanobrevibacter* was dominating, and the group 2500 and 1000 μm were lower than group 100 and 10 μm (*P*<0.001). Furthermore, the relative abundance of genera *Methanosphaera* of group 2500 μm was greater than the other three groups (*P*<0.001), and group 1000 μm was greater than group 100 and 10 μm (*P*<0.001).

**Table 8:** Effect of alfalfa meal particle size on cecum archaeal populations in rabbits.

**Parameter**	**Particle size (μm)**				**SEM**	*P* **value**
	2500	1000	100	10		
g__*Methanobrevibacter* (%)	62.48^c^	75.93^b^	89.68^a^	90.40^a^	2.419	<0.001
g__*Methanosphaera* (%)	35.47^a^	23.04^b^	8.39^c^	8.26^c^	2.392	<0.001
g__*Methanothermobacter* (%)	1.53	0.32	0.35	0.51	0.187	0.054
g__*Methanobacterium* (%)	0.34	0.01	0.34	0	0.098	0.438
g__Unclassified (%)	0	0.001	0.002	0	0.001	0.381

^a,b,c^Different superscripts in the same row indicate significant differences (*P*≤0.05).

## Discussion

### Growth performance

The results of the present study showed that rabbits in 2500 μm group reached 90.6~91.5% of the final live weight of the other three treatment groups, and the ADFI and ADG values of 1000, 100 and 10 μm groups were greater than 2500 μm group. The increase in the final weight of the treatment groups indicated that the growing rabbits were able to more efficiently utilize diets with finer particle size. Previous studies investigated the effect of dietary fiber particle size on the performance of rabbits [10,35], but in contrast to the present study, none reported a influence of fineness of grinding on growth traits, possibly because the particle size of the fiber used in these studies was greater than 1.5 mm. Reimer [36] noted that particle size is the second factor that would dictate approximately 20% of pellet quality; smaller particles will have a greater number of contact points within a pellet matrix compared to larger ones, so a smaller particle size will lead to greater granulation hardness. Rabbits must keep grinding their teeth for their w hole lives, prefering to chew hard granules, so when the hardness of the granules increased, the feed intake casually increased. However, no differences were observed by Sogunle et al. [37], who used fiber particle sizes of 1 and 2 mm and ground all the other ingredients to the same sizes; only the particle size of the alfalfa meal was changed in the current study.

Gidenne [6] observed that particles <0.3 mm were retained in the cecum longer than particles >0.3 mm, and this result was further supported by a later study found that an increase in the proportion of fine particles increased cecal retention time and fiber digestion efficiency but decreased dry matter intake [9]. The inconsistency might be caused by different hardness of the diets.

### Nutrient digestibility and metabolism

The alfalfa meal particle size had a marked effect on the apparent digestibility of CP, NDF and ADF. In terms of feed efficiency, data from different trials carried out with feedstuffs containing particles of different sizes exhibit discrepancies [12,38]. It is obvious the digestibility of fiber will increase as the particle size decreases, and reducing the particle size can increase the surface area so that the fiber has more interaction sites with microbial digestive enzymes. This has also been reported in other species, such as pigs [39–40]. However, the highest digestibility of crude protein was observed in the 1000 μm group, and this result was mirrored by the small intestine morphology that will be discussed later. A similar trend in apparent crude protein digestibility was reported by Tufarelli et al [12], who found that rabbits fed diets with particle sizes of 1 mm had greater protein digestibility than diets with particle sizes of 2 mm. The protein content of alfalfa meal is as high as 20%, and the increased digestibility of CP in the alfalfa meal promoted the digestibility of the diet.

In this experiment, the protein concentrations of the feeds were designed the same in each group, however, the CP content increased as the alfalfa hay particlce size decreased. The increase in IN and GEI also led to an increase in FN, FE,UN and UE, which are the two main pathways for the loss of ingested nutrients. A similar tendency was previously observed in growing pigs in which a greater daily protein intake resulted in an increase in FN and UN [41]. It is speculated that the crude protein (16%) used in the present experimental diets is at almost the highest concentration among recommendations worldwide, so the protein utilization efficiency could not be further promoted. The enhanced growth performance might be partly attributed to the increased feed intake.

### Cecal environment

The pH of the cecal content indicates the extent of cecal fermentation and is negatively correlated with the diarrhea rate in rabbits [42]. No difference in pH was observed in the present study, which might have resulted from the fiber content being sufficient for the rabbits (NDF, 30%). On the other hand, the pH value is a combined indicator of the total concentration of VFA and NH_3_-N. Although there were no variations in total VFA, the decreased NH_3_-N led to a minor decrease in pH value (5.99 vs. 5.81). Furthermore, the decreased NH_3_-N may reflect more efficient NH_3_-N utilization by the cecal microbes. Carabaño et al. [43] reported that increased availability of a fermentable substrate could promote microbial protein synthesis, thus reducing the NH_3_-N concentration in the cecum, but no difference was observed in the production of MCP. In addition, molar proportion of acetic acid and propionic acid (% TVFA) were increased while the butyric acid was decreased. García et al. [44] observed a influence of the particle size of fiber ingredients in the feed on the molar proportion of short-chain fatty acids, but Nicodemus et al. [2] demonstrated that a reduction in dietary particle size had no effect on the molar proportions of acetic, propionic and butyric acids. The reason may be that the gradient of decreasing particle size was not sufficiently large.

### Small intestinal and gastric morphology

Gut morphology is an indicator of intestinal health and absorption capability. An extended villus length can expand the total villi absorption area, and subsequently lead to more digestive enzymes and greater nutrient digestibility on the surface of the villi [45]. In the present study, the greatest villus height and the villus height: crypt depth ratio of the jejunum and ileum were obtained in 1000 μm group. The alfalfa ground through a 1.0-mm sieve lengthened the villi, thus increasing the villus height:crypt depth ratio. This intestinal characteristic was consistent with the CP digestibility discussed above and consistent with Sogunle et al. [36], who reported improved intestinal morphology when rabbits were fed diets with a particle size of 1 mm compared with 2 mm. Romero et al. [11] also reported that finely ground (1.5 mm), dehydrated alfalfa increased the villous height and decreased the crypt of jejunum when compared with grinding at 4.5 mm. Desantis et al. [46] reported that rabbits fed fine particles (2 mm) displayed more irregularly shaped, greater duodenal villi and deeper crypts in the distal colon as well as a greater number of goblet cells than rabbits fed coarse (8 mm) particles. The superfine grinding of fibers (100 and 10 μm) had a somewhat detrimental effect on small intestine morphology in present study. All of the measurements, including villus height, decreased; crypt depth increased; and the villus height:crypt depth ratio decreased. Degeneration and necrosis of mucosal epithelial cells, gastric inflammatory cell infiltration, and muscular myometrial edema were also observed more frequently in the stomach. Therefore, there must be an appropriate particle size for the development of the small intestine; too coarse or too fine particles are harmful to the intestinal and stomach morphology.

### CH_4_ emission and archaeal populations

Our study found that the NDF digestibility increased, and the methane production increased with the reduction of alfalfa particle size. Previous research has shown that NDF digestibility influences rumen fermentation characteristics and the passage rate [47–48], so forages with greater degradability will result in more intensive fermentation in the rumen [48], thereby increasing TVFA production, this was also observed in present study. On the other hand, Johnson et al. [49] reported that CH_4_ emissions account for 3–12% of the GE intake in cattle, and the results of this study showed that CH_4_ emission from the finest particle size group was approximately 0.2% of the GE intake. Rabbits emit little methane. Apart from CH_4_, 30–40% of the maintenance energy requirement of adult rabbits could be covered by the microbial fermentation products of short-chain fatty acids [50–51]. Microbial fermentation in the rabbit cecum resembles rumen fermentation in ruminants. In adult rabbits, there are approximately 10^9^−10^11^·g^-1^ cells in the cecal contents, which harbor bacteria and methanogenic archaea [52]. In the current study, the diversity of methanogens decreased with the decrease in alfalfa meal particle size and Witzig et al. [53] reported that ruminant microbial community structure changed according to feed particle size. However, Zhu et al. [17] reported no significant differences in the diversity and abundance of caecal archaeal community of rabbits that were fed diets with different dietary fibre-to-starch ratios.

*Methanobrevibacter* and *Methanosphaera* are two dominant H_2_-consuming organisms that are usually found in the rumen and hindgut [54]. *Methanobrevibacter* produces one mole of methane per mole of carbon dioxide [55], while *Methanosphaera* requires four moles of methanol to produce three moles of CH_4_ [56]. This means that the ability of *Methanobrevibacter* to produce methane is stronger than that of *Methanosphaera*. The abundance of *Methanobrevibacter* was promoted while that of *Methanosphaera* was decreased when the particle size of the alfalfa meal decreased. This might lead to increased CH_4_ production when the alfalfa meal particle size is decreased. However, the mechanisms warrant further investigation.

## Conclusions

Better growth performance can be obtained in rabbits through fine grinding of fiber ingredient, although this has a detrimental effect on the gastro-intestinal morphological development. Reducing the particle size of dietary fiber can decrease archaeal community diversity as well as increase the abundance of cecal *Methanobrevibacter* at the expense of *Methanosphaera*. This variation in methanogens subsequently promotes methane production, however the methane energy accounts for only approximately 0.2% of the gross energy intake of rabbits, which is negligible compared with that documented in other ruminants.

## Supporting information

S1 Table Measurement of particle of ground alfalfa. (DOCX)

S1 Fig Typical rabbits jejunum and ileum slice from 2500 (A1,2),1000 (B1,2),100 (C1,2) and 10 (D1,2) μm (HE staining, 40 X) group respectively.
(TIF)

S2 Fig Typical rabbits gastric tissue section from 2500(A),1000(B),100(C) and 10 (D) μm (HE staining, 400 X) group respectively.
(TIF)

S3 Fig Rarefaction curves of the cecal archaeal communities. Rarefaction curves of operational taxonomic units (OTUs) were calculated at the 97% level of similarity.
(TIF)

S4 Fig Heatmap of genus abundance in the 24 rabbit cecal samples. A hierarchical dendrogram showing the distribution of bacteria across the 24 rabbit cecal samples. Different colors indicate different relative values for the archaeal genera, and the legend is presented at the top of the figure.
(TIF)
