# Supplementary material for: Effects of the particle of ground alfalfa hay on the growth performance, methane production and archaeal populations of rabbits
Source: PLoS One. 2018 Sep 17;13(9):e0203393. doi: 10.1371/journal.pone.0203393 (PMC6141101; doi:10.1371/journal.pone.0203393)
Supplement: S1 Table — (DOCX) [file pone.0203393.s001.docx]

S1 Table. Measurement of particle size.

| Plan particle size（μm） | 2500 | 1000 | 100 | 10 |
| --- | --- | --- | --- | --- |
| Average sizes (μm) | 466.35 | 222.91 | 83.24 | 25.98 |
| Specific area (m^2^/g) | 0.92 | 1.13 | 1.53 | 2.15 |
