# Supplementary figures and images for: Effects of the particle of ground alfalfa hay on the growth performance, methane production and archaeal populations of rabbits
Source: PLoS One. 2018 Sep 17;13(9):e0203393. doi: 10.1371/journal.pone.0203393 (PMC6141101; doi:10.1371/journal.pone.0203393)

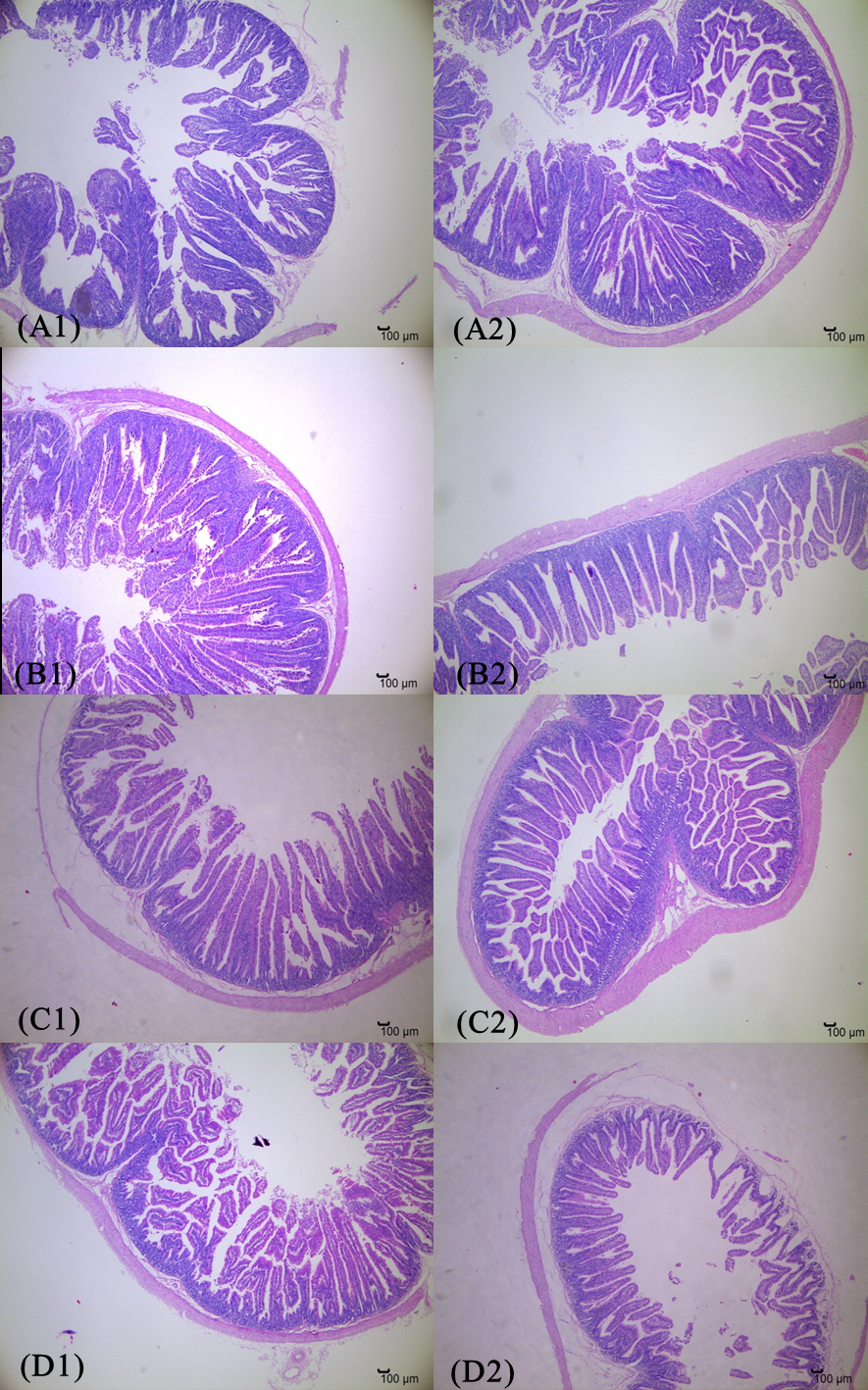

Supplement: S1 Fig — Typical rabbits jejunum and ileum slice from 2500 (A1,2),1000 (B1,2),100 (C1,2) and 10 (D1,2) μm (HE staining, 40 X) group respectively. (TIF) [file pone.0203393.s002.tif]

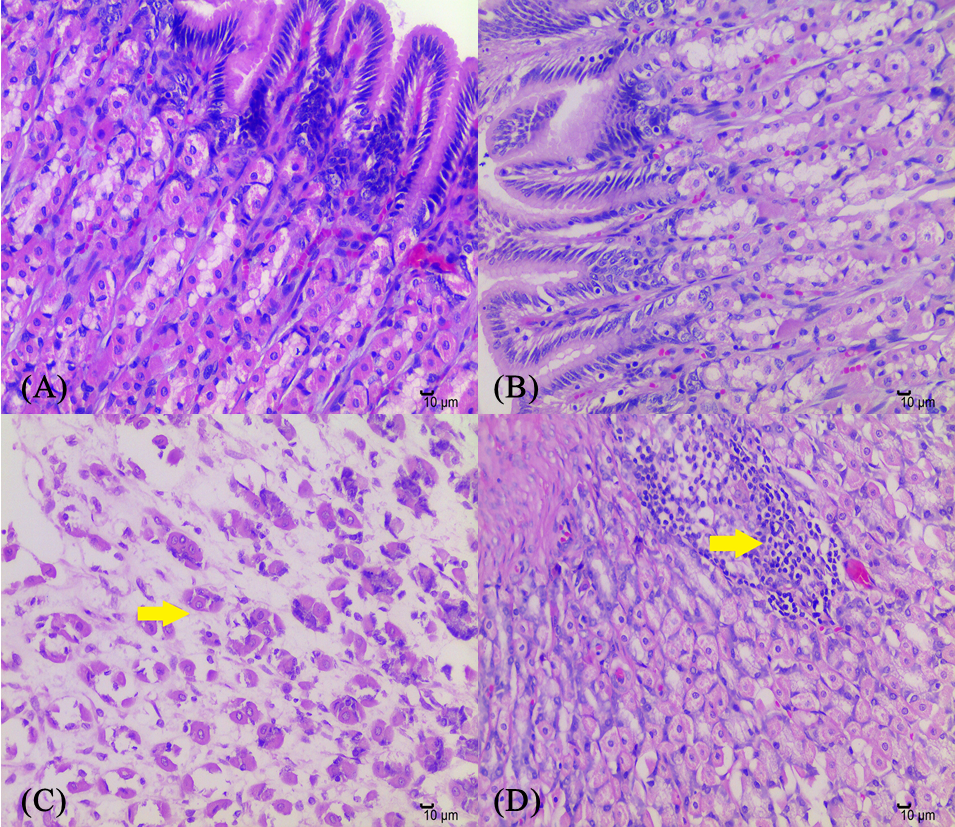

Supplement: S2 Fig — Typical rabbits gastric tissue section from 2500(A),1000(B),100(C) and 10 (D) μm (HE staining, 400 X) group respectively. (TIF) [file pone.0203393.s003.tif]

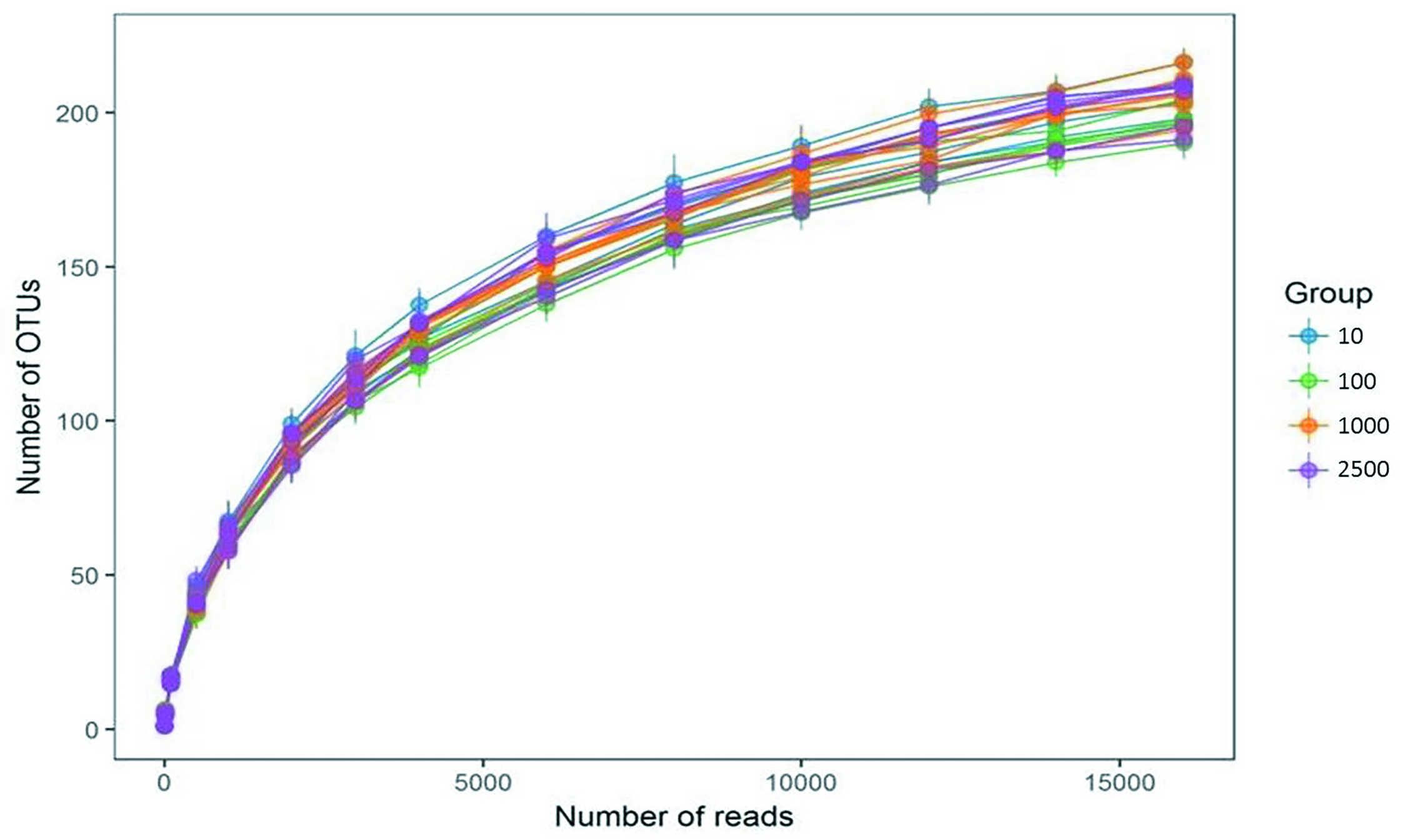

Supplement: S3 Fig — Rarefaction curves of operational taxonomic units (OTUs) were calculated at the 97% level of similarity. (TIF) [file pone.0203393.s004.tif]

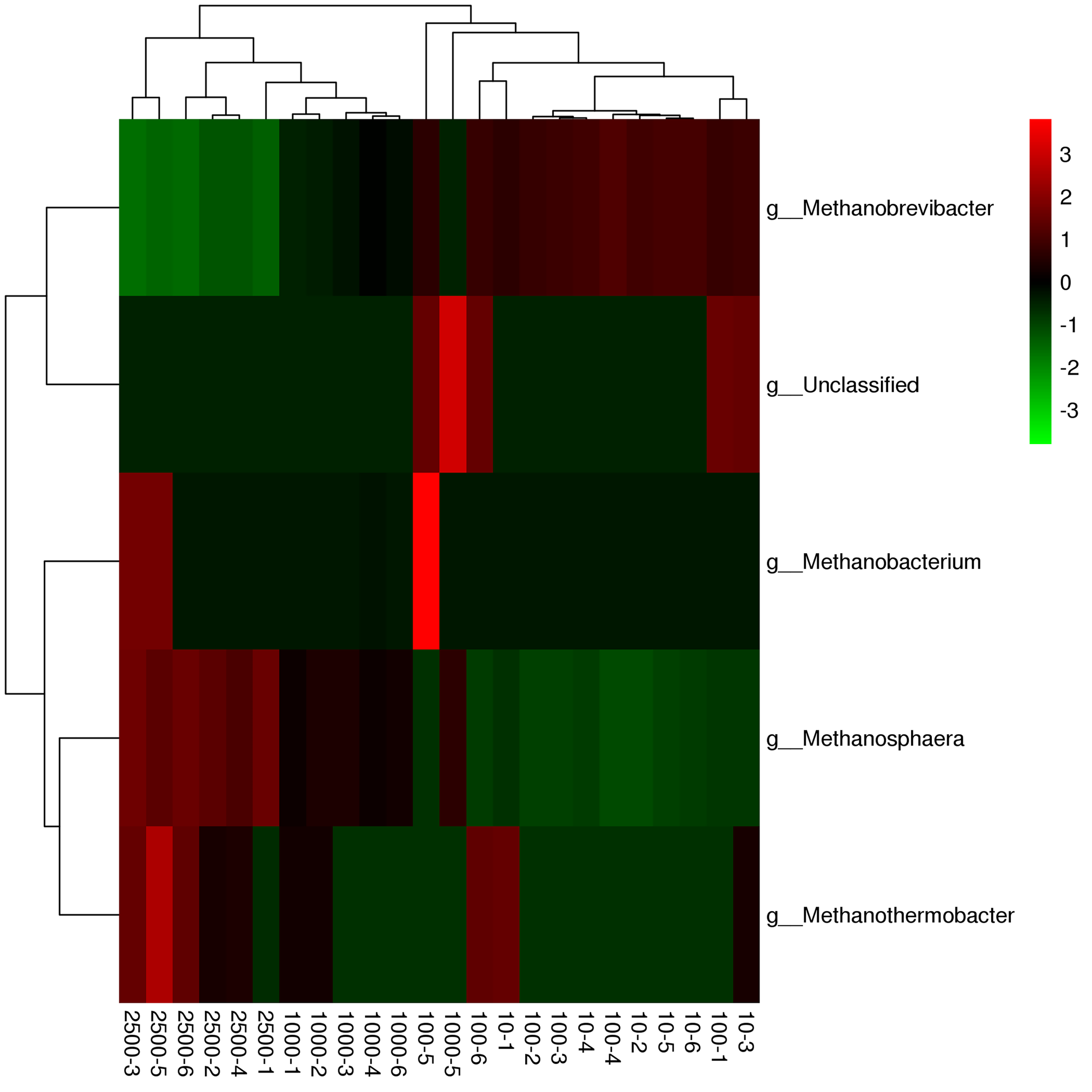

Supplement: S4 Fig — A hierarchical dendrogram showing the distribution of bacteria across the 24 rabbit cecal samples. Different colors indicate different relative values for the archaeal genera, and the legend is presented at the top of the figure. (TIF) [file pone.0203393.s005.tif]
